# Serum Hemoglobin Level, Anemia, and Growth Were Unaffected by a 12-Month Multiple-Micronutrient Powder Intervention Among Children Aged 8–10 Months in a Low-Socioeconomic-Status Community of Jakarta

**DOI:** 10.3390/nu17152520

**Published:** 2025-07-31

**Authors:** Dian Novita Chandra, Saptawati Bardosono, Tonny Sundjaya, Tjhin Wiguna, Rini Sekartini

**Affiliations:** 1 Department of Nutrition, Faculty of Medicine Universitas Indonesia, Dr. Cipto Mangunkusumo General Hospital, Jakarta 10430, Indonesia; 2 Human Nutrition Research Center, Indonesian Medical Education and Research Institute (HNRC IMERI), Faculty of Medicine, Universitas Indonesia, Jakarta 10430, Indonesia; 3 Department of Epidemiology, Faculty of Public Health, Universitas Indonesia, Depok 16424, Indonesia; s_ton77@yahoo.com; 4 Department of Psychiatry, Faculty of Medicine Universitas Indonesia, Dr. Cipto Mangunkusumo General Hospital, Jakarta 10430, Indonesia; twiga00@yahoo.com; 5 Department of Child Health, Faculty of Medicine Universitas Indonesia, Dr. Cipto Mangunkusumo General Hospital, Jakarta 10430, Indonesia; rsekartini@yahoo.com

**Keywords:** anemia, growth, hemoglobin level, infants 8–10 months, multi-micronutrient powder

## Abstract

**Background and Objectives**: Anemia and stunting are major public health concerns for young Indonesian children. Limited information is available from studies on multi-micronutrient supplements in Indonesia. The objective of this study was to investigate the effect of 12-month multi-micronutrient powder (MNP) supplementation on serum hemoglobin levels, anemia, and growth. **Methods:** A randomized double-blinded placebo-controlled study was performed, including 232 eligible children aged 8–10 months old. Children with severe anemia or stunting/those classed as underweight were not included as subjects. The study was performed in a low-socioeconomic-status community in Jakarta. With an active-to-placebo ratio of 60:40, 139 subjects received MNP sachets twice/day, and 93 subjects received placebo sachets, mixed with complementary food. The outcome parameters were hemoglobin level, anemia, and growth indicators. Per-protocol analysis was performed for 179 (intervention: 110; control: 69) subjects. **Results:** There were no differences at baseline between the groups, except for the weight-for-length z-scores (WLZ). Upon intervention, the serum hemoglobin level and anemia proportion did not change significantly within the group, and no significant differences were found between the groups (*p* > 0.05). However, subgroup analysis of non-anemic children at baseline showed a significant increase in hemoglobin levels in the youngest age group (8.0–8.9 months old) receiving MNP compared to placebo (0.13 vs. −0.79, *p* = 0.031). Iron deficiency anemia proportion showed a similar upward trend upon intervention in both groups. No significant differences in growth were found between both groups. **Conclusions:** This study failed to find a significant effect of 12-month MNP supplementation on serum hemoglobin level, anemia, and growth.

## 1. Introduction

In populations where anemia is a public health problem, the point-of-use fortification of complementary foods with iron-containing micronutrient powders (MNPs) in infants and young children aged 6–23 months is recommended, in order to improve iron status and reduce anemia (WHO, 2016: strong recommendation, moderate-quality evidence) [1]. A prevalence of anemia of 20% or higher in infants and young children under 2 years of age is considered a public health problem. A trend assessment of the prevalence of anemia among Indonesian children, adolescents, women, and men from the Indonesia Family Life Surveys from 1997 to 2008 showed a clear decreasing trend. In the 2008 survey, an anemia prevalence of 31% in children under five was reported, meaning the public health concern was still considered as moderate [2]. In a smaller survey pertaining to the 2009–2010 period, in a low-socioeconomic-status (SES) community in Jakarta, we found hemoglobin (Hb) levels below 11 mg/dL in 57% of 6–8-month-old infants, alongside suboptimal cognitive adaptive test scores, prompting an urgent call for the provision of iron-rich complementary foods [3].

The World Health Organization (WHO) guidelines on the use of multi-micronutrient powders (MNPs) are based on an updated version of the 2011 Cochrane review, which includes high-quality evidence regarding the reduction in anemia and low- and very low-quality evidence regarding the increase in hemoglobin levels, improvement of iron status, and improvement of weight-for-age (in z-score) [1,4]. Systematic reviews from 2020 have strengthened this evidence base, confirming that MNP supplementation significantly reduces both overall anemia prevalence (RR 0.76, 95% CI 0.69–0.84) and iron deficiency anemia (RR 0.45, 95% CI 0.34–0.58), while noting an associated increase in diarrhea incidence (RR 1.30, 95% CI 1.11–1.53) [5]. The 2016 guideline suggested a scheme for point-of-use fortification of foods with MNPs consumed by infants and young children aged 6–23 months which includes equivalents of 10–12.5 mg elemental iron (e.g., equivalent to 37.5 mg ferrous fumarate) and 5 mg of elemental zinc and vitamin A (300 mcg, as retinol) optionally combined with other micronutrients to achieve 100% of the Recommended Nutrient Intake (RNI) [1]. However, amongst the 12 relevant studies reviewed by Salam et al., studies with higher dosages of iron and zinc (up to 30 and 10 mg, respectively) and studies with vitamin A levels ranging from 100 to 400 mg retinol equivalent are worthy of consideration [6].

In Indonesia, in 2006, the Ministry of Health initiated a program to design and implement an MNP under the project and product name Taburia. The composition of this MNP (10 mg iron, 5 mg zinc, 417 mcg vitamin A, and other micronutrients) is in line with the WHO guideline [7]. Several efficacy and effectiveness studies have been performed, and it was found that Taburia with ferrous fumarate is equally effective compared to Taburia with sodium-iron-EDTA [8]. Some projects have commented on the limited compliance and poor sensory characteristics of the products [9,10,11,12]. Large-scale introduction of Taburia was achieved in 2013, having been under consideration for several years.

The WHO guidelines on recommended dietary allowance for children aged 1–3 years were higher than the actual Indonesian children dietary requirement. Concern was expressed about the excess burden of diarrhea and respiratory morbidity associated with MNP treatment in several populations with malnourished children and high diarrhea burdens [6,13]. A plausible explanation for association between the increase in diarrhea and iron-containing MNPs in some studies was found after assessment of the effects on the gut microbiota, where excess colonic iron can stimulate the growth and virulence of many pathogenic enterobacteria [14]. An example of a safer mode for preventive and therapeutic provision of iron to infants and young children living in infectious environments may be a hepcidin-based screen-and-treat approach [15]. Therefore, the development of a novel strategy of micronutrient supplementation for children in Indonesia is highly important.

The objective of the present study was to provide representative information on the effectiveness of using MNPs with iron contents according to Indonesian children’s dietary requirements (80% of WHO guidelines) among young children aged 8–10 months. Younger infants (8–10-month olds) typically consume semi-solid foods, enabling practical MNP adherence and practical implementation, ensuring full 12-month intervention completion before 23 months. We aimed to evaluate whether locally tailored MNPs can effectively address anemia and iron deficiency at this vulnerable stage. Our primary outcome parameter was hemoglobin level at 12 months of intervention, and the secondary outcomes included anemia prevalence and iron deficiency anemia based on serum ferritin (corrected for C-reactive protein) and growth.

## 2. Materials and Methods

### 2.1. Study Design and Ethics

This study was a randomized, controlled, double-blinded, paralleled-group, single-centered exploratory study that was conducted in the slum–urban subdistricts of Cempaka Putih, Senen (central part of Jakarta), and Jatinegara (eastern part of Jakarta). This study was conducted by following good clinical practice (GCP), taking place between started February 2014 and February 2016. The study was approved by the Medical Ethics Committee, Faculty of Medicine Universitas Indonesia (Approval No. 680/H2.F1/ETIK/2012) and is registered in the ClinicalTrials.gov PRS database as NCT01840384.

### 2.2. Subjects and Randomization

Sample size calculation was performed using the SAS Syntax program (Version 9.3) and was based on the hemoglobin concentration as the primary endpoint parameter. Assuming a mean difference of 0.5 g/dL at follow-up and SD = 1.12 g/dL [16,17], a total sample size of 167 young children would be required for a statistical power of 80%, using an alpha of 0.05 and two-sided *t*-test with a 60:40 active-to-placebo group ratio (100:67). Due to ethical considerations aimed at minimizing the number of participants receiving placebo, an unbalanced allocation ratio of 3:2 (active/placebo) was employed. The sample size was calculated accordingly to maintain adequate statistical power under this allocation scheme and ensure efficient use of study resources. Although a 2:1 ratio was considered, this would have required a larger total sample size, which was not feasible within the study’s logistical and financial constraints. Assuming a dropout rate of 30%, the recruitment target for the study was set at 217 (130:87) to ensure a statistical power suitable for a planned per-protocol analysis.

Inclusion criteria included apparently healthy infants aged 8–10 months at recruitment and living in the study area. Written informed consent was obtained from the parents before the screening process but after the investigators had explained the study to each parent. Exclusion criteria were Hb level < 8 g/dL, severe undernutrition (indicated by either length-for-age z-scores (LAZ) < −3SD, weight-for-age z-scores (WAZ) < −3SD, or weight-for-length z-scores (WLZ) < −3SD), history of premature birth or low birth weight (<2500 g), congenital abnormality or any other known conditions likely to affect nutrient absorption, disorders requiring special diet, and any other chronic illnesses or conditions which could have interfered with the assessment. A general physical examination was performed at the screening stage to determine eligibility. Relevant aspects of patients’ medical history and other pre-existing conditions were recorded. Medical history was defined as any medical event that occurred after birth until the date of informed consent. Any medical condition with which the subject was born, e.g., congenital abnormality/malformations, was recorded as a pre-existing condition. Infants with severe anemia or malnutrition require immediate treatment, and low-birth-weight or premature infants are at risk of both iron deficiency and overload; therefore, they were not included as subjects.

The local health cadres assisted in the recruitment of subjects at the community-integrated service posts (Posyandu) in the study area. A total of 290 children were screened for their eligibility. Randomization was conducted at the baseline visit, which occurred within 30 calendar days following the screening visit, after the confirmation of participant eligibility. A total of 232 eligible subjects were randomly assigned in a 139:93 ratio to receive either the investigational product (intervention group) or placebo (control group), administered as two sachets per day for 12 months.

The allocation sequence was generated using a computer-based block randomization method to ensure balanced group sizes throughout the enrolment period. The block size was fixed (details withheld to preserve allocation concealment), and the sequence was stratified to maintain group balance. To implement the random allocation sequence, sequentially numbered, identical packaging was prepared and labeled according to the randomization list by an independent statistician not involved in participant enrolment or assessment. The allocation sequence was concealed from investigators and participants until the point of assignment to prevent selection bias. The coding was unblinded after all participants completed the intervention period, when all data were entered. Due to several reasons, 29 subjects from the intervention group and 24 subjects from the control group discontinued the study; thus, only 179 subjects (intervention: 110; control: 69) completed the study and were included in the per-protocol analysis.

### 2.3. Intervention

In the current study, a privately developed MNP (2 sachets/day providing 8 mg iron as ferrous fumarate, 8 mg zinc, 400 mcg vitamin A, and other micronutrients) was investigated according to a double-blinded placebo-controlled design. Regarding the composition of the MNP for the intervention group, as shown in Table 1, it consists of several vitamins and minerals needed for growth and the prevention and alleviation of anemia. The product was developed and produced only for this study. The product was given in two servings per day to fully meet the WHO guideline for MNPs for zinc and vitamin A and 80% of the minimal content for iron. The levels of other minerals and micronutrients were targeted to provide between 60 and 100% of the Indonesian RNI for children aged 6–12 months and/or 1–3 years of age [18]. The placebo product for the control group consisted of maltodextrin only. Both the MNP and placebo sachets had similar appearances and were labeled with five different codes (MNP to placebo = 3:2) to ensure the blinding process.

The investigators and trained study staff gave detailed instructions (verbal and written) on how to consume the MNP (i.e., sprinkled and mixed into complementary feeding food to be consumed by the subjects (two sachets/day)). The parents were asked to keep and fill in a diary of each serving consumption and keep empty sachets, to be reviewed by the investigators on each new visit. The study staff counted the empty sachets and compared them with the diary to determine adherence.

The subjects were requested not to consume other supplements like syrup/multi-micronutrient supplements or iron supplementation for more than 3 months. Subjects were excluded from the study in cases of severe growth faltering/undernutrition occurring during the intervention period, insufficient use of study products (<40 sachets in the last 4 weeks), regular use of other supplement syrup/multi-micronutrients (every day in >1 week), and/or the need for iron supplementation (>3 months). Excluded subjects were not replaced, and their subject numbers were not reassigned.

### 2.4. Data Collection and Outcome Measures

**Figure 1:**
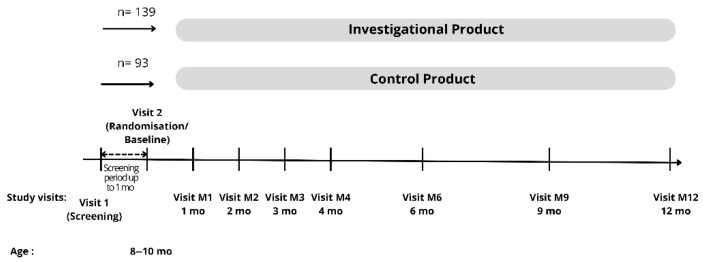
Data collection.

This study included 9 visits for each subject: the screening visit, baseline/randomization (T0), 1 month, 2 month, 3 month, 4 month, 6 month (T1), 9 month, and endline (12 month (T2)). Visits within 3 days before or after the appointment date were tolerated for the 1- to 4-month visits, and visits 7 days before or after the appointment date were tolerated for the 6-month–12-month visits. Figure 1 summarizes the overall data collection visits on each subject.

#### 2.4.1. Anthropometric Assessments

Anthropometric measurements were performed at screening, baseline, and each visit up to the endline (12 month visit) by using standardized techniques. Undressed infants were weighed on an electronic scale (Seca 354, Seca Gmbh & Co. Kg, Hamburg, Germany) with an accuracy of 10 g. Recumbent length was measured to the nearest 1 mm. The measurements were performed by trained dietitian study staff who were experienced with the method, and the same calibrated pieces of equipment (Seca 417, Seca Gmbh & Co. Kg, Hamburg, Germany) were used for each visit. At any time, 3 measurements were taken for each growth parameter, and the average value was considered. Actual chronological decimal age was used to calculate the standardized anthropometric indices (z-scores). The z-scores represented the distance in standard deviation (SD) units from reference growth values based on age and gender (according to the WHO 2006 growth charts) [19].

#### 2.4.2. Subjects’ Medical Information

Physical examination was also conducted on every visit, and the occurrence of any illness/medical symptoms throughout the study was reported by the parents to the study staff. Any deterioration of a pre-existing condition and/or clinically significant elements of one’s medical history found during the study period were reported as an adverse event (AE). All physical examinations were conducted by the pediatrician study staff.

#### 2.4.3. Biochemical Assessments

At the first visit for screening, finger prick blood sampling to determine hemoglobin level was performed by trained study staff using HemoCue^®^ Hemoglobin systems (HemoCue AB, Ängelholm, Sweden) [20]. Venous blood samples were collected at baseline (T0), month 6 of the intervention (T1), and at the end of the intervention (12 month or T2) to determine the hemoglobin, C-reactive protein (CRP), and ferritin levels [21]. Blood sample analyses were performed by an internationally accredited laboratory (Prodia Clinical Laboratory, Jakarta, Indonesia). Subjects with CRP-levels of ≥10 mg/L were excluded from the analyses for serum ferritin levels. Iron deficiency proportion were calculated based on the cut-off value of ferritin < 12 mcg/L. The values pertaining to the proportion of anemia were calculated based on the WHO cut-off value of Hb < 11.0 g/dL and iron deficiency anemia (IDA) with an additional criterion of serum ferritin < 12 μg/L.

#### 2.4.4. Complementary Feeding Practice and Other Questionnaires

Dietary intake was assessed using the single 24 h food recall at baseline (T0; when the subjects were aged 8–10 months old), at the 6 month (T1; when the subjects were aged 14–16 months old), and at the end of the intervention (T2; when the subjects were aged 20–22 months old) by trained dietitians (study staff).

At the 3-, 6-, 9-, and 12-month visits, parents in both groups were given the same nutrition education flyers on macronutrient intake since the study product needed to be mixed in with complementary feeding food. Data on continued breastfeeding and the dietary diversity of the complementary feeding practice were taken from the 24 h food recall. Continued breastfeeding was reported as a percentage. The dietary diversity score of complementary feeding was reported based on the aggregated feeding practices for eight food groups: (1) breastmilk; (2) grains, roots, tubers, and plantains; (3) legumes, pulses, and nuts; (4) dairy products; (5) flesh foods; (6) eggs; (7) vitamin A-rich fruits–vegetables; and (8) other fruits–vegetables. Minimum dietary diversity was defined as achieved if the subject consumed at least 5 of the 8 food groups [22].

Data on the subject’s demographic, socioeconomics, and family characteristics at baseline were collected using a validated questionnaire created by the Indonesian Ministry of Health (Indonesian Demographic and Health Survey 2012 questionnaire) [18].

### 2.5. Statistical Analysis

All data were analyzed using the Statistical Package for Social Science (SPSS) version 20.0. The normality of the data was analyzed using the Kolmogorov–Smirnov test. Normally distributed data are presented in mean ± SD format, while not normally distributed data are presented as median values (interquartile ranges). Delta (Δ) is the calculated difference between the baseline and endline numerical data (T2 minus T0) within each group. Differences between the intervention groups were analyzed by using an independent *t*-test or the Mann–Whitney U-test, depending on the normality of the data. Comparison between groups for categorical data was conducted by using a chi-square test or Fisher’s Exact test. A value of *p* < 0.05 was considered statistically significant.

## 3. Results

Over 12 months, this study evaluated the effect of multi-micronutrient powder (MNP) provided to children from the age of 8–10 months on hemoglobin level, anemia, and growth.

**Figure 2:**
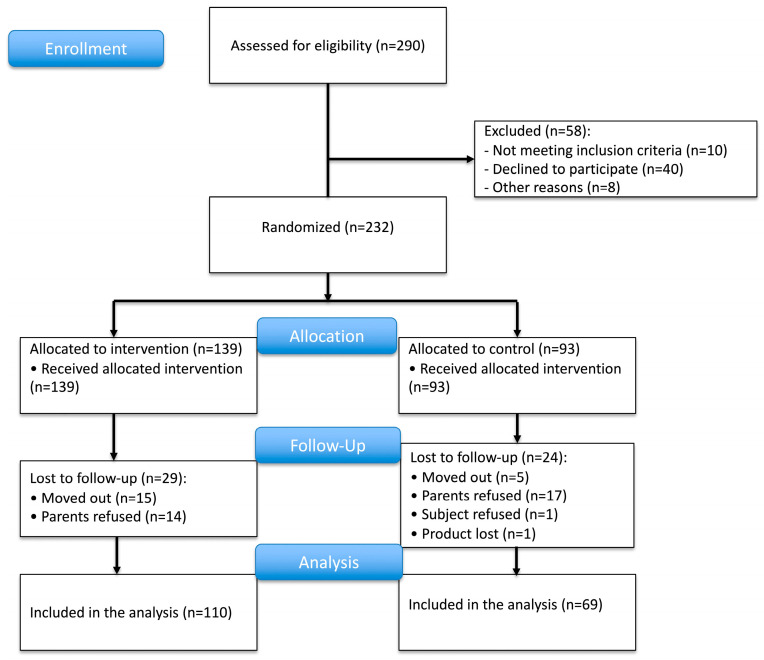
Trial profile.

As shown in Figure 2, we recruited 232 subjects from 290 subjects screened in the study area. This number was slightly higher than the 217 subjects required according to the power analysis as we were not fully sure about the assumed dropout rate of 30%. The subjects were randomly allocated into the intervention and control groups at a ratio of 60:40. Thus, 139 and 93 subjects were included in each group, respectively. Non-eligible children with Hb levels < 8 g/dL and/or severe undernutrition at screening were referred to obtain immediate treatment. During the 12-month intervention period, 29 and 24 subjects from the intervention and control group dropped out from the study, respectively. Finally, we were able to analyze 110 subjects in the intervention group and 69 subjects in the control group in the per-protocol analysis. The number of subjects was sufficient according to the sample size calculation.

The main characteristics for illustrating the subject’s characteristics and parent’s social status are presented in Table 2.

There were no differences in characteristics between the groups. The subjects belonged to reproductive-age parents, being mostly from a small family with two or fewer children. Approximately 60% of mothers had high educational status, i.e., >nine years of formal schooling, but approximately 90% only completed household chores. More than 20% of fathers had no permanent job. A very high percentage of smoking habits within at least one family member (>80%) was recorded, which is typical for low-socioeconomic-status communities in Indonesia.

Table 3 shows that there was no significant difference in continued breastfeeding practice and minimum dietary diversity at baseline and during the intervention period. Breastfeeding practices were slightly better among the intervention group; however, the difference with the control group was not statistically significant. The increase in dietary diversity score during the intervention was higher in the control group.

The length-for-age z-scores (LAZ), weight-for-age z-scores (WAZ), and weight-for-length z-scores (WLZ) in both groups were equal at baseline (*p* > 0.05, independent *t*-test). No significant changes were found in the WAZ and the status of WAZ (underweight) during the 12-month intervention period. LAZ decreased in both the intervention and control groups during the intervention period. Consequently, the stunted status (LAZ < −2SD) of the children increased significantly between the baseline measurement and after the 12-month intervention. As a randomization bias, the status of the WLZ < −2SD was significantly higher in the control group at baseline (*p* < 0.05; chi-square test). No significant differences were found in the delta of WAZ, LAZ, and WLZ between the two groups during the 12-month intervention period.

Table 4 shows the main outcome-related measurements of our study: hemoglobin (Hb) and serum ferritin at baseline, at 6 and 12 months of intervention. A small number of blood samples could not be analyzed due to lysis. Hb levels remained fairly constant during the intervention period, and there was also no difference between groups. Subjects with CRP-levels ≥ 10 mg/L were excluded from the analysis for serum ferritin levels. This affected between 5 and 20% of the samples per time point per group. It can be seen from Table 4 that ferritin levels decreased within each group upon the period of intervention. There were no statistically significant differences comparing the subject’s ferritin levels between the control and intervention groups of the delta and at any time point.

**Figure 3:**
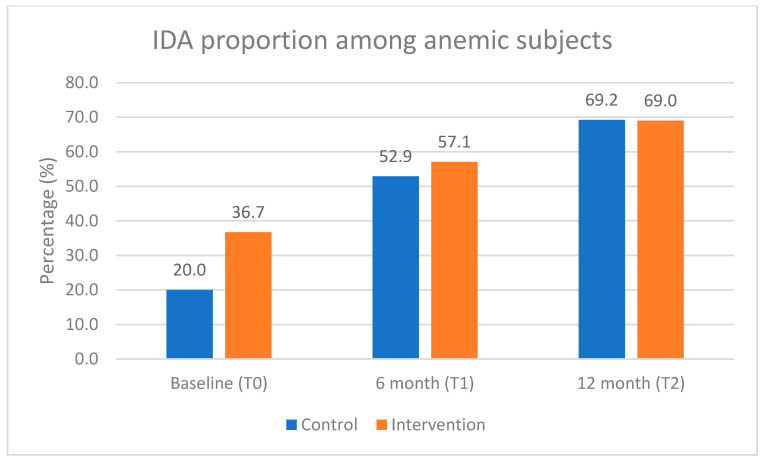
Proportion of iron deficiency anemia (IDA) among anemic subjects during the intervention period.

There was a slight improvement in overall anemia in the control group but not in the intervention group. However, this trend was not statistically significant. Iron deficiency anemia (IDA) increased during the intervention period in both groups. At baseline (infant age 8–10 months), IDA accounted for 20% and 36.7% of overall anemia in the control and intervention groups, respectively, while at the end of the study, IDA made up more than 2/3 in both groups (69.2% vs. 69.0%), as shown in Figure 3.

**Figure 4:**
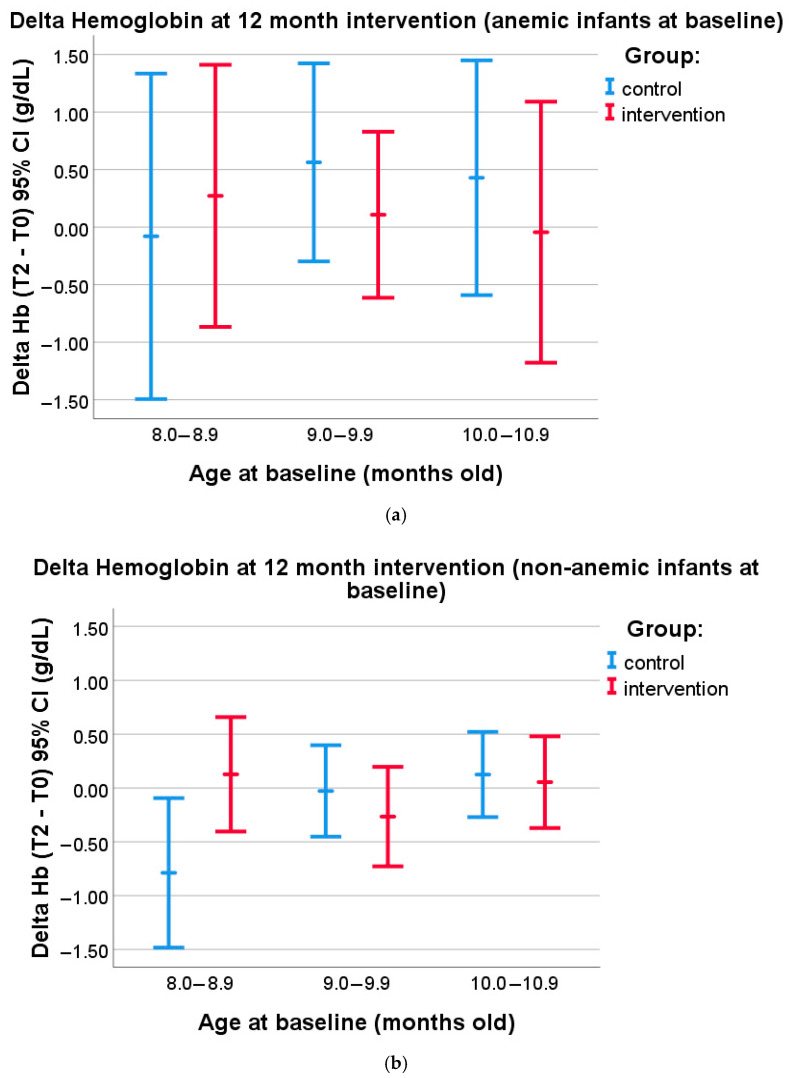
(**a**) Subgroup analysis in anemic subjects at baseline (8.0–8.9 months old: control *n* = 5, intervention = 7; 9.0–9.9 months old: control *n* = 8, intervention *n* = 14; and 10.0–10.9 months old: control *n* = 7, intervention *n* = 9). Delta of Hemoglobin levels at 12 month intervention in the subjects belonging to either the control or the intervention group, segmented according to age at baseline (mean values and 95% confidence intervals are given). (**b**) Subgroup analysis in non-anemic subjects at baseline (8.0–8.9 months old: control *n* = 9, intervention *n* = 22; 9.0–9.9 months old: control *n* = 18, intervention *n* = 32; and 10.0–10.9 months old: control *n* = 20, intervention *n* = 24). Delta of Hemoglobin levels at 12 month intervention in the subjects belonging to either the control or the intervention group, segmented according to age at baseline (mean values and 95% confidence intervals are given).

A post hoc subsample analysis was carried out to investigate whether different effects of the MNP intervention could be seen in subjects of different ages who were anemic or non-anemic at baseline. The nutrient level in the MNPs was enough to fulfill daily requirements; thus, the main benefit is more related to prevention. Therefore, we classified the subjects into non-anemic and anemic subjects. We further classified the subjects according to their age at baseline (8.0–8.9 months old—12 anemic subjects and 31 non-anemic subjects; 9.0–9.9 months old—22 anemic subjects and 50 non-anemic subjects; and 10.0–10.9 months old—16 anemic subjects and 44 non-anemic subjects) to find out whether the subjects receiving the supplement earlier in life would benefit more in terms of iron deficiency prevention. We tested, for each age group within both the anemic and the non-anemic subgroups, whether there was a difference in the delta of hemoglobin at 12 months of the intervention period between the control and intervention groups. Figure 4a shows that, in the subgroup of anemic subjects at baseline, no differences were found in the delta of Hb levels in each age group in both the intervention and the control groups (*p* > 0.05, independent *t*-tests). However, in the non-anemic subgroup, a statistically significant difference in the delta of Hb level was found among the youngest age group (8.0–8.9 months old) between the control and intervention groups (−0.79 CI: −1.5 to −0.1 vs. 0.13 CI: −0.4 to 0.6, *p* = 0.031; independent *t*-test) (Figure 4b). Detailed analysis results can be seen in Supplementary Table S1.

## 4. Discussion

This randomized, double-blinded, placebo-controlled trial evaluated the impact of 12-month multiple micronutrient powder (MNP) supplementation on hemoglobin levels, anemia prevalence, and growth among children aged 8–10 months in a low-socioeconomic-status community in Jakarta. The findings revealed no significant differences between the intervention and control groups for the primary outcomes, providing nuanced insights into the effectiveness of MNP supplementation in this context.

The intervention did not significantly affect hemoglobin levels or anemia prevalence, consistent with several previous studies highlighting the variable efficacy of MNPs across different populations and settings [23,24,25]. While anemia prevalence slightly declined in the control group, this trend was absent in the intervention group. Additionally, iron deficiency anemia (IDA) increased in both groups, particularly around the age of 12 months in our population, eventually comprising over two-thirds of all anemia cases by the study’s conclusion. This increase may indicate the challenges in effectively addressing iron deficiency intervention through current MNP formulations or dietary practices.

We performed several additional analyses and, in this paper, report results according to our subject classification, based on age at the start of supplementation and the separation of anemic and non-anemic subjects. This revealed a significant difference in the delta of hemoglobin levels among non-anemic children in the youngest age group (8.0–8.9 months old) and highlighted the decrement in the control group, which was not found in the intervention group, suggesting a potential beneficial effect of MNPs in terms of the prevention of anemia in this subset. This discrepancy may be due to differences in complementary feeding recommendations for infants older than 8 months compared to those aged 6–8 months, which may result in alterations to their dietary patterns [22]. This would mean that, in our population, only the youngest children, uncomplicated by anemia, may have had a direct benefit from MNP according to our protocol. For all the other (sub)groups, there might be other factors which we did not control for preventing any significant effect on our outcome parameters. Kounnavong et al. [26] also conducted a subgroup analysis, dividing moderate and severe anemic, mild anemic, and non-anemic children, but they did not segment according to age as well. Over the full 24-week treatment period, the change in Hb level between the control and treatment groups was statistically significant in the non-anemic and the mildly anemic group but not in the moderate and severe anemic subjects. Unfortunately, the age range of their study population was rather broad from 6 to 52 months, which makes comparison with our study difficult. In a recent systematic review and meta-analysis of 21 studies on MNP supplementation in children from low- and middle-income countries, it was found that MNP use was clearly associated with a lower risk of anemia. Interestingly, subgroup analyses according to age at baseline between studies showed a non-significant trend in response between anemic and non-anemic children in favor of older (12–23 months) compared to younger children (6–11 months) [5]. On the other hand, Andrew et al. [27] speculate that the micronutrient content of the diet, particularly during the early weaning period, is crucial to prevent anemia. The ineffectiveness and likely inefficacy of MNP supplementation among 12–24-month-old children in Colombia could be attributed to dietary diversity and micronutrient intake after the age of 12 months not contributing greatly to high rates of early childhood anemia in their population.

Recent studies have highlighted the variable efficacy of micronutrient powder (MNP) supplementation across different populations and settings. For instance, a randomized controlled trial in rural Bangladesh found that MNP supplementation did not significantly affect hemoglobin levels or anemia prevalence among children aged 2–5 years, even in areas with high groundwater iron levels. These findings suggest that MNP supplementation may not significantly impact hemoglobin levels or anemia prevalence in certain contexts, particularly in populations with adequate iron intake from other sources, such as groundwater. Similarly, a cluster-randomized trial in rural China reported that while MNP supplementation led to modest improvements in hemoglobin levels, it did not significantly reduce the prevalence of anemia among young children. These findings underscore the need for context-specific interventions that consider the nutritional status, infection burden, and dietary practices of target populations to effectively combat anemia [23,24].

The rapid pace of early life growth and development demands substantial iron intake. At the same time, young children—with their still-developing immune systems—are especially susceptible to infections by iron-dependent pathogens [28]. In our study, almost all subjects experienced at least one episode of infection during the 12-month intervention, with no differences between the intervention and control groups. Upper respiratory tract infection was the most common infection among both groups. Diarrhea was more common in the intervention group, even though this factor was not significantly different (detailed analysis results can be found in Supplementary Table S2). Thus, the iron content in the supplementation is possibly not related with the infection episodes.

Iron plays a vital role in hemoglobin synthesis, facilitating efficient oxygen transport to body tissues. Adequate oxygenation is fundamental to cellular proliferation and developmental growth. Additionally, iron levels modulate the activity of Insulin-like Growth Factor 1 (IGF-1), a key regulator of growth. Insufficient iron has been associated with diminished IGF-1 function, particularly in children undergoing rapid developmental phases. Studies found that iron supplementation has been shown to enhance growth only in cases where children are affected by iron deficiency anemia [29].

The absence of significant changes in growth parameters, such as WAZ, LAZ, and WLZ, aligns with findings from other studies indicating that standalone MNP interventions have limited effects on growth. For instance, a cluster-randomized controlled trial in rural Burkina Faso found that while MNP supplementation, combined with nutrition education, marginally improved growth among children aged 6–23 months, the effects were limited. This suggests that standalone MNP interventions may have limited effects on growth [30]. Stunting and growth faltering are influenced by a combination of factors, including dietary diversity, maternal nutrition, and infection burden. Similarly, a systematic review and meta-analysis on MNP supplementation in children from low- and middle-income countries reported that, while MNP use was associated with a lower risk of anemia, the effects on growth outcomes were often negligible unless paired with broader nutritional and environmental interventions [5].

This study’s strengths include its robust design and focus on a high-risk, low-resource population, providing critical insights into the potential and limitations of MNP interventions. By using a 60:40 ratio for the intervention and control groups, we planned to give more children the perceived benefit of the intervention. Additionally, we felt an ethical obligation to provide all study participants with nutritional education. However, several limitations must be acknowledged. Anemia, in this study population, is likely influenced by multifactorial causes, including iron deficiency, infections, chronic inflammation, and genetic hemoglobin disorders. Additionally, environmental factors such as heavy metal poisoning, especially lead poisoning, are a common public health problem in Jakarta, potentially contributing to iron-resistant anemia and limiting the efficacy of iron-based interventions like MNPs. While iron supplementation can benefit lead-exposed children, this complex interaction can manifest as iron-resistant anemia, warranting specific consideration in future nutritional interventions and study designs [31,32,33,34].

Compared to our earlier study [3], baseline Hb and anemia proportion at baseline clearly improved, and the educational status of the mothers in our study was rather good (60%: high level), indicating progress at several levels in our recruitment area. It may even be conceivable that the effect of nutritional education was stronger than any effect of the multi-micronutrient supplement. This aligns with the findings of Somasse et al. [25], which highlight the importance of integrating MNPs with broader interventions, such as nutrition education, to address the complex determinants of anemia and malnutrition. Together, these findings emphasize the need for context-specific, multifaceted approaches that go beyond micronutrient supplementation to include education, dietary improvements, and infection control, particularly in settings where socioeconomic and environmental factors play a significant role in health outcomes. However, this remains a hypothesis requiring verification through studies specifically designed to isolate these effects.

The MNP used in this study met Indonesian children’s RNI for the targeted age group but provided only 80% of the recommended iron dosage, according to WHO recommendations. Evidence suggests that higher iron doses (10–12.5 mg/day) may yield better anemia reduction outcomes in infants and children aged 6–23 months [35]. While our MNP was designed to address common micronutrient deficiencies, its composition may not have optimally met the nutritional needs of the target population. For instance, Barffour et al. [36] conducted a randomized controlled trial in rural Laos and found that daily MNP supplementation had a limited impact on reducing anemia and improving growth outcomes among children, despite improvements in certain micronutrient biomarkers. This suggests that, while MNPs may address specific micronutrient deficiencies, their overall effectiveness in improving anemia and growth may be constrained by contextual factors such as infection burden, dietary diversity, and baseline nutritional status. If the formulation lacked certain key micronutrients or was not tailored to the specific deficiencies prevalent in this demographic, the effectiveness of the intervention could be limited. Tailored formulations with optimized nutrient balances may enhance the outcomes. While some Indonesian studies have reported positive effects of MNP supplementations (Taburia) containing 10 mg of elemental iron, in accordance with 80% of the recommended iron dosage of the WHO guidelines, in malnourished populations, our null results in healthy infants suggest important limitations and changes in dietary/nutritional recommendations [7,37].

Despite the convincing evidence from the large systematic reviews and meta-analyses on the effectiveness and efficacy of multiple-micronutrient powder supplements, many studies have failed to demonstrate the expected effects [27,38]. Various factors may have contributed to this failure, including the composition of the supplements not being fully suitable for the target population or environmental factors like high iron groundwater [23]. Considering these insights, it is imperative to consider the unique characteristics and contextual factors of the target population when designing micronutrient interventions. Tailored approaches are essential not only for enhancing adherence but also for optimizing the effectiveness of micronutrient powder (MNP) supplementation. The Editorial “Micronutrient Powders for Infants and Young Children”, based on a study in Rwanda, highlights the significance of providing comprehensive support to improve adherence and uptake, which is crucial for maximizing the benefits of MNP programs. This study emphasizes that interventions aimed at enhancing logistics and adherence strategies are key to reducing anemia prevalence among children [39]. Therefore, further research into the factors influencing compliance and the overall efficacy of MNP supplementation is necessary to refine public health strategies and improve nutrition and health outcomes for young children.

## 5. Conclusions

In conclusion, this study highlights the limited impact of 12-month MNP supplementation on hemoglobin levels, anemia prevalence, and growth outcomes among healthy infants in a low-socioeconomic setting. These findings underscore the importance of context-specific strategies, suggesting that (1) future interventions may require age-targeted formulations; (2) MNP efficacy could be enhanced through reformulations addressing local micronutrient deficits (e.g., higher bioavailable iron); and (3) combined approaches integrating MNPs with systematic infection control, dietary diversification programs, and supervised nutrition education may yield greater benefits than standalone supplementation.

## Figures and Tables

**Table 1:** Composition of multi-micronutrient powder (study product).

Nutrient ^a^	Unit	Amount/Day (5 g) ^b^
Calcium (as calcium carbonate)	mg	480
Iron (as ferrous fumarate)	mg	8
Zinc (as zinc sulfate)	mg	8
Iodine (as potassium iodate)	mcg	90
Selenium (as sodium selenite)	mcg	11
Vitamin A (as retinol acetate)	mcg	400
Vitamin D3 (as cholecalciferol)	mcg	5
Vitamin E (as dl-alpha-tocopheryl acetate)	IU	6
Vitamin B1 (as thiamine HCl)	mg	0.5
Vitamin B2 (as riboflavin)	mg	0.5
Vitamin B6 (as pyridoxine)	mg	0.4
Vitamin B12 (as cyanocobalamin)	mcg	0.6
Vitamin C (as sodium ascorbate)	mg	40
Folic Acid	mcg	90
Niacin (as niacinamide)	mg	5
Pantothenic Acid	mg	2

^a^ maltodextrin was used as a filling agent. ^b^ 2.5 g per sachet.

**Table 2:** Subjects’ and parents’ social characteristics. Data for the control and intervention groups are shown.

Variables	Control (*n* = 69)	Intervention (*n* = 110)	*p*-Value
Child’s gender (%)			
Boys	50.7	48.2	0.740
Girls	49.3	51.8	
Age			
Child (mo)	9.1 ± 1.1 ^†^	8.9 ± 0.9 ^†^	0.140
8.0–8.9 months, %	20.3	27.3	
9.0–9.9 months, %	39.1	40.8	0.357
10.0–10.9 months, %	40.6	31.9	
Mother (y)	28.5 ± 6.1 ^†^	28.8 ± 5.9 ^†^	0.710
Father (y)	31.9 ± 7.0 ^†^	33.4 ± 6.7 ^†^	0.155
Number of children (%)			
≤2	71.0	63.6	0.309
>2	29.0	36.4	
Educational level (%)			
Mother			
Low	43.4	39.1	0.561
High	56.6	60.9	
Occupational status (%)			
Mother			
Outside the house	10.1	10.0	0.713
In house	89.9	90.0	
Father			
Not permanently	27.5	22.8	0.077
Permanent job	72.5	77.2	
Smoking habit in family members (%)	84.1	80.9	0.593

educational level low—up to junior high school; educational level high—senior high school and above. ^†^, mean ± SD; statistical analysis: independent *t*-test for age; others: chi-square test.

**Table 3:** Minimum dietary diversity and anthropometric indicators of children before and during 12 months of MNP supplementation.

Variables	Control (*n* = 69)	Intervention (*n* = 110)	*p*-Value	Mean Difference (95% CI)
Continued Breastfeeding (%)				
Baseline (T0)	85.5	93.6	0.071 ^a^	
6 months (T1)	81.2	83.6	0.670 ^a^	
12 months (T2)	55.1	67.3	0.101 ^a^	
Dietary diversity score				
Baseline (T0)	4.0 (1.0) ^d^	4.0 (1.0)	0.110 ^b^	
6 months (T1)	5.0 (1.0)	5.0 (1.0)	0.640 ^b^	
12 months (T2)	5.0 (1.5)	5.0 (1.0)	0.165 ^b^	
Delta (T2 − T0)	2.0 (2.0)	1.0 (2.0)	0.049 ^b^	
Minimum dietary diversity (%)				
Baseline (T0)	17.4	20.0	0.665 ^a^	
6 months (T1)	58.0	54.5	0.653 ^a^	
12 months (T2)	66.7	56.4	0.170 ^a^	
LAZ				
Baseline (T0)	−0.5 ± 1.1 ^e^	−0.5 ± 1.0	0.647 ^c^	
6 months (T1)	0.7 ± 1.1	−0.8 ± 1.0	0.356 ^c^	
12 months (T2)	−0.9 ± 1.1	−1.0 ± 1.1	0.587 ^c^	
Delta (T2 − T0)	−0.4 ± 0.9	−0.5 ± 0.8	0.885 ^c^	0.0 (−0.2–0.3)
WAZ				
Baseline (T0)	−1.2 ± 0.8	−1.0 ± 1.0	0.231 ^c^	
6 months (T1)	−1.3 (1.3)	−1.2 (1.3)	0.968 ^b^	
12 months (T2)	−1.0 ± 1.1	−1.0 ± 1.0	0.937 ^c^	
Delta (T2 − T0)	0.2 ± 0.8	−0.1 (0.9)	0.072 ^b^	
WLZ				
Baseline (T0)	−1.2 ± 1.0	−0.9 ± 1.0	0.135 ^c^	
6 months (T1)	−1.2 ± 1.0	−0.9 ± 1.0	0.154 ^c^	
12 months (T2)	−0.8 ± 1.0	−0.7 ± 1.1	0.390 ^c^	
Delta (T2 − T0)	0.4 ± 0.9	0.2 (0.9)	0.120 ^b^	
LAZ < −2SD (%)				
Baseline (T0)	5.8	5.5	0.923 ^a^	
6 months (T1)	8.7	10.0	0.772 ^a^	
12 months (T2)	17.4	18.2	0.893 ^a^	
WAZ < −2SD (%)				
Baseline (T0)	15.9	18.2	0.700 ^a^	
6 months (T1)	15.9	17.3	0.817 ^a^	
12 months (T2)	20.3	17.3	0.612 ^a^	
WLZ < −2SD (%)				
Baseline (T0)	23.2	11.8	0.044 ^a^	
6 months (T1)	15.9	12.7	0.546 ^a^	
12 months (T2)	11.6	11.8	0.964 ^a^	

LAZ: length-for-age z-score; WAZ: weight-for-age z-score; WLZ: weight-for-length z-score; T0: baseline; T1: at 6 month of intervention; T2: at 12 month of intervention; delta: within-group T2 minus T0. ^a^ chi-square test, ^b^ Mann–Whitney U test, ^c^ independent *t*-test. ^d^ median (interquartile range), all such values, ^e^ mean ± Standard Deviation, all such values.

**Table 4:** Hemoglobin, ferritin, anemia, and iron status of children at baseline (T0), at 6- (T1) and 12-month (T2) intervention periods.

Variables	Control (*n* = 69)	Intervention (*n* = 110)	*p*-Value	Mean Difference (95% CI)
Hemoglobin (g/dL)				
Baseline (T0)	11.6 ± 1.1 ^d^	11.5 ± 1.1	0.694 ^a^	0.1 (−0.3–0.4)
6 months (T1)	11.4 ± 1.5	11.4 ± 1.1	0.992 ^a^	0.0 (−0.4–0.4)
12 months (T2)	11.6 ± 1.1	11.5 ± 1.4	0.555 ^a^	0.1 (−0.3–0.5)
Delta (T2 − T0)	0.0 ± 1.0	0.0 ± 1.2	0.806 ^a^	0.0 (−0.3–0.4)
Ferritin (µg/L)				
Baseline (T0)	27.1 (45.3) ^e^ (*n* = 57)	32.7 (48.7) (*n* = 93)	0.403 ^b^	
6 months (T1)	26.6 (39.7) (*n* = 53)	30.0 (39.4) (*n* = 104)	0.550 ^b^	
12 months (T2)	22.2 (21.9) (*n* = 64)	20.6 (26.3) (*n* = 103)	0.686 ^b^	
Delta (T2 − T0)	−7.9 (32.2) (*n* = 53)	−10.2 (32.2) (*n* = 91)	0.324 ^b^	
Anemia (%)				
Baseline (T0)	29.4	27.8	0.815 ^c^	
6 months (T1)	25.8	25.7	0.992 ^c^	
12 months (T2)	19.1	26.4	0.269 ^c^	
Iron deficiency (%)				
Baseline (T0)	18.9 (*n* = 57)	16.5 (*n* = 93)	0.638 ^c^	
6 months (T1)	18.0 (*n* = 53)	18.9 (*n* = 104)	0.954 ^c^	
12 months (T2)	24.5 (*n* = 64)	30.8 (*n* = 103)	0.476 ^c^	
Iron deficiency anemia (%)				
Baseline (T0)	7.5 (*n* = 57)	11.0 (*n* = 93)	0.393 ^c^	
6 months (T1)	14.0 (*n* = 53)	13.3 (*n* = 104)	0.962 ^c^	
12 months (T2)	15.1 (*n* = 64)	18.7 (*n* = 103)	0.560 ^c^	

T0: baseline; T1: at 6 month of intervention; T2: at 12 month of intervention; delta: within-group T2 minus T0. ^a^ independent *t*-test, ^b^ Mann–Whitney U test, ^c^ chi-square test. ^d^ mean ± standard deviation, all such values, ^e^ median (interquartile range), all such values.

## Data Availability

The raw data supporting the conclusions of this article will be made available by the authors on request.

## Supplementary Materials

The following supporting information can be downloaded at https://www.mdpi.com/article/10.3390/nu17152520/s1, Table S1: Hemoglobin changes at 12 month intervention among anemic and non-anemic subjects; Table S2: Infection morbidity among subjects during the intervention.
