# Supplementary material for: Serum Hemoglobin Level, Anemia, and Growth Were Unaffected by a 12-Month Multiple-Micronutrient Powder Intervention Among Children Aged 8–10 Months in a Low-Socioeconomic-Status Community of Jakarta
Source: Nutrients. 2025 Jul 31;17(15):2520. doi: 10.3390/nu17152520 (PMC12348989; doi:10.3390/nu17152520)
Supplement: Supplementary file 1 [file nutrients-17-02520-s001.zip › nutrients-3749511-supplementary.pdf]

Supplementary Table S1 Hemoglobin changes at 12 month intervention among anemic and non-anemic subjects

Variables	Control (mean ± SD)	Intervention (mean ± SD)	p-value	Mean difference (95% CI)
Anemic subjects	n=20	n=30		
8.0 - 8.9 months old	n=5	n=7		
Hb baseline (T0), g/dL	10.4 ± 0.5	10.6 ± 0.2	0.440 [a]	-0.2 (-0.7 - 0.3)
Hb 6 month (T1), g/dL	10.1 ± 1.7	10.3 ± 1.1	0.810 [a]	-0.2 (-1.9 - 1.5)
Hb 12 month (T2), g/dL	10.4 ± 1.3	10.9 ± 1.3	0.493 [a]	-0.5 (-2.2 - 1.1)
Delta Hb (T2-T0), g/dL	-0.1 ± 1.1	0.3 ± 1.2	0.626[a]	-0.3 (-1.9 - 1.2)
9.0 – 9.9 months old	n=8	n=14		
Hb baseline (T0), g/dL	10.4 ± 0.3	10.2 ± 0.5	0.325 [a]	0.2 (-0.2 – 0.6)
Hb 6 month (T1), g/dL	10.9 ± 1.9	10.7 ± 1.1	0.747 [a]	0.2 (-1.1 – 1.5)
Hb 12 month (T2), g/dL	11.0 ± 1.1	10.3 ± 1.3	0.248 [a]	0.7 (-0.5 – 1.8)
Delta Hb (T2-T0), g/dL	0.5 ± 1.0	0.1 ± 1.2	0.393[a]	0.4 (-0.6 - 1.5)
10.0 – 10.9 months old	n=7	n=9		
Hb baseline (T0), g/dL	10.3 ± 0.5	10.1 ± 0.7	0.595 [a]	0.2 (-0.5 – 0.9)
Hb 6 month (T1), g/dL	10.9 ± 0.9	10.1 ± 1.2	0.217 [a]	0.7 (-0.5 – 1.9)
Hb 12 month (T2), g/dL	10.7 ± 1.3	10.3 ± 1.2	0.566 [a]	0.4 (-1.0 – 1.7)
Delta Hb (T2-T0), g/dL	0.4 ± 1.1	-0.0 ± 1.5	0.492[a]	0.5 (-1.0 - 1.9)
Non-anemic subjects	n=47	n=78		
8.0 - 8.9 months old	n=9	n=22		
Hb baseline (T0), g/dL	12.3 ± 1.3	11.7 ± 0.4	0.237 [a]	0.6 (-0.4 - 1.6)
Hb 6 month (T1), g/dL	10.9 ± 2.5	11.7 ± 0.9	0.404 [a]	-0.7 (-2.6 - 1.2)
Hb 12 month (T2), g/dL	11.5 ± 1.4	11.8 ± 1.3	0.520 [a]	-0.3 (-1.4 - 0.7)
Delta Hb (T2-T0), g/dL	-0.8 ± 0.9	0.1 ± 1.2	0.031[a]	-0.9 (-1.8 - 0.0)
9.0 – 9.9 months old	n=18	n=32		
Hb baseline (T0), g/dL	12.0 ± 0.7	12.1 ± 0.9	0.721 [a]	-0.1 (-0.6 - 0.4)
Hb 6 month (T1), g/dL	11.7 ± 0.5	11.9 ± 0.9	0.407 [a]	-0.2 (-0.6 - 0.3)
Hb 12 month (T2), g/dL	12.0 ± 0.7	11.8 ± 1.4	0.573 [a]	0.2 (-0.5 - 0.2)
Delta Hb (T2-T0), g/dL	0.0 ± 0.8	-0.3 ± 1.3	0.486[a]	0.2 (-0.4 - 0.9)
10.0 – 10.9 months old	n=20	n=24		
Hb baseline (T0), g/dL	12.1 ± 0.7	12.2 ± 0.9	0.813 [a]	-0.0 (-0.5 - 0.4)
Hb 6 month (T1), g/dL	12.1 ± 0.9	11.9 ± 0.9	0.361 [a]	0.2 (-0.3 - 0.8)
Hb 12 month (T2), g/dL	12.2 ± 0.8	12.2 ± 0.8	0.963 [a]	0.0 (-0.5 - 0.5)
Delta Hb (T2-T0), g/dL	0.1 ± 0.8	0.0 ± 1.0	0.804[a]	0.1 (-0.5 - 0.6)

[a] independent t-test

Supplementary Table S2. Infection morbidity among subjects during the intervention

Variables	Control (n=69)	Intervention (n=110)	p-value
Upper respiratory tract infection			
Morbidity (%)	89.9	90.9	0.815[a]
Number of episodes	2.0 (3.0)[d]	3.0 (2.0)	0.301[b]
Diarrhea			
Morbidity (%)	18.8	30.9	0.074[a]
Number of episodes	0.0 (0.0)	0.0 (1.0)	0.108[b]
Skin infection			
Morbidity (%)	43.5	56.4	0.093[a]
Number of episodes	0.0 (1.0)	1.0 (1.0)	0.148[b]
Eye infection			

Morbidity (%)	1.4	7.3	0.156[c]
Number of episodes	0.0 (0.0)	0.0 (0.0)	0.084[b]
Unspecified fever			
Morbidity (%)	49.3	36.4	0.088[a]
Number of episodes	0.0 (1.0)	0.0 (1.0)	0.195[b]
Viral infection			
Morbidity (%)	4.3	4.5	1.000[c]
Number of episodes	0.0 (0.0)	0.0 (0.0)	0.950[b]
Any infection			
Morbidity (%)	97.1	96.4	1.000[c]
Number of episodes	4.0 (4.0)	4.5 (3.0)	0.186[b]

[a] chi-square test, [b] Mann-Whitney U test, [c] Fisher Exact test

[d] Median (Inter Quartile Range), all such values
